# Dosimetric and Radiobiological Evaluation of Inhomogeneity-Corrected Dose Distribution in Prophylactic Radiotherapy for Heterotopic Ossification

**DOI:** 10.3390/jcm14155291

**Published:** 2025-07-26

**Authors:** Than S. Kehwar, Indra J. Das

**Affiliations:** Department of Radiation Oncology, Northwest Memorial Hospital, Northwestern University Feinberg School of Medicine, Chicago, IL 60611, USA; than.kehwar@nm.org

**Keywords:** radiation treatment, inhomogeneity correction, heterotopic ossification, radiobiological consequence

## Abstract

**Background/Objectives:** The aim of this study was to evaluate the impact of inhomogeneity correction (IC) of dose distribution on the dosimetric and radiobiological efficacy of radiation treatment for heterotopic ossification (HO). **Methods:** This study involved a retrospective analysis of 21 patients treated using a homogeneous dose distribution plan for hip prophylactic HO. These IC-off plans were evaluated against an IC-on dose distribution plan. Dosimetric and corresponding radiobiological parameters (gEUD, LQ-EUD, LQ, EQD2 for α/β = 3 and 10 Gy) were calculated. These parameters were compared for both treatment plans. Additionally, Monte Carlo simulations were performed using mean and standard deviation values from baseline data to generate 10,000 synthetic datasets, allowing for robust statistical modeling of variability in dose distributions and biological outcomes. **Results:** The homogeneous (IC-off) plans demonstrated overestimation of dose conformity and uniformity, reflected in lower HI values (0.10 ± 0.05 vs. 0.18 ± 0.05) and higher D_90%_–D_98%_ coverage. Radiobiologically, these plans yielded higher gEUD (7.02 Gy vs. 6.80 Gy) and EQD2 values across all α/β scenarios (e.g., EQD2_[α/β=3]_gEUD_ = 14.07 Gy vs. 13.35 Gy), with statistically significant differences (*p* < 0.001). Although IC-on plans demonstrated steeper dose gradients (higher GIs), this came at the expense of internal dose variability and potentially compromised biological effectiveness. **Conclusions:** Our results suggest that plans without IC deliver suboptimal biological effectiveness if continued preferentially in routine HO prophylaxis. With advanced radiation dose calculation algorithms available in all centers, inhomogeneity-corrected doses warrant prospective validation.

## 1. Introduction

Heterotopic pathophysiology refers to the presence or development of tissues, organs, or cells in locations where they are not normally found; in some instances, they form heterotopic ossifications with a crystalline composition that is similar to that of normal bone, with both containing hydroxyapatite as the primary mineral. This suggests that the mineralization process in heterotopic ossifications is like that of bone formation in normal skeletal structures [1]. This phenomenon can occur because of developmental abnormalities, trauma, or various pathological conditions, leading to the formation of ectopic tissue in unusual anatomical locations. While heterotopy can be a benign and incidental finding in some cases, it often results in debilitating consequences, especially when it involves tissues such as bone, cartilage, or glandular structures. Heterotopic ossification (HO), a hallmark of heterotopic pathophysiology, is the formation of bone in soft tissues, including muscles, tendons, and ligaments. It is most commonly observed following trauma or surgery, especially in patients with spinal cord injuries or traumatic brain injuries or those undergoing hip repair or replacement surgery [2,3]. It can also occur in individuals with conditions such as burn injuries or neurological disorders, such as cerebral palsy [4].

The underlying molecular mechanisms that drive HO are complex and multifactorial. Inflammatory cytokines, growth factors, and signaling pathways such as bone morphogenetic proteins (BMPs), transforming growth factor-beta (TGF-β), and wingless-related integration site (Wnt) signaling have been shown to play pivotal roles in the initiation and progression of HO [5]. The term “Wnt” was created by combining “wingless” (a Drosophilia gene) and “Int-1” (a mouse gene). Wnts form a large family of secreted protein growth factors that play a crucial role in regulating cell growth, motility, and differentiation, particularly during embryonic development. In adults, Wnts have a role in homeostasis, and inappropriate activation of the Wnt pathway is implicated in a variety of neoplastic conditions [6]. Furthermore, the role of mesenchymal stem cells (MSCs) in ectopic bone formation is increasingly being recognized. These cells, under specific conditions, can differentiate into osteoblasts and contribute to the aberrant mineralization that characterizes HO [7]. Importantly, while the formation of heterotopic bone may seem benign, it can lead to significant functional impairment, pain, and compromised mobility, particularly when it involves large joints or nerve compression. As a result, the management of these conditions has become a major focus in orthopedic and rehabilitation medicine.

In addition to HO, heterotopy may also occur in other tissues, leading to pathological conditions such as heterotopic endometriosis, ectopic pancreas, and heterotopic thyroid tissue [8]. These ectopic tissues often cause dysfunction by interfering with normal organ function or by eliciting a chronic inflammatory response that can lead to fibrosis or obstruction. The challenge for clinicians lies in the recognition of these rare pathologies, which requires a high degree of suspicion and advanced diagnostic techniques, such as imaging modalities and histopathological analysis.

Given the often debilitating nature of heterotopic conditions, effective therapeutic strategies are essential. Among these, radiotherapy (RT) has emerged as a promising non-invasive approach, particularly in the prevention and treatment of HO [9]. The primary mechanism through which radiotherapy impacts HO formation is through the inhibition of osteogenesis, where ionizing radiation suppresses the differentiation of progenitor cells into osteoblasts, thus preventing abnormal bone formation in soft tissues [10]. The use of low-dose RT, typically administered as a single fraction of 7–8 Gy, has proven effective in reducing the incidence and severity of HO in high-risk patients [11,12,13,14]. Radiotherapy is particularly beneficial when administered early post-operatively, typically within 72 h following surgery or injury, to prevent the formation of HO at sites of trauma or surgical intervention.

Recent advancements have focused on optimizing the timing, dosage, and delivery methods of radiotherapy to achieve better therapeutic outcomes while minimizing potential side effects. A key challenge in radiotherapy for heterotopic ossification lies in determining the optimal radiation dose. While doses between 7 and 8 Gy have been effective at preventing HO in clinical trials [15], variations in patient response suggest that personalized treatment regimens tailored to individual risk factors are needed. Moreover, advancements in 3D conformal radiotherapy (3D-CRT) and intensity-modulated radiotherapy (IMRT) have allowed for more precise targeting of the radiation beam, minimizing exposure to surrounding healthy tissues and reducing potential complications such as skin damage, ulceration, or secondary malignancies [16,17]. In recent years, radiation therapy with advanced technologies has significantly progressed to enable accurate dose prescription with inhomogeneity correction.

Despite the promising outcomes of radiotherapy in the management of heterotopic conditions, several challenges remain. The timing of radiotherapy administration remains a critical factor, with the most effective outcomes achieved when treatment is delivered early in the disease course [18]. Additionally, the long-term risks associated with ionizing radiation, including the potential for secondary malignancies, remain a concern, particularly in younger patients and those with a history of radiation exposure [19]. Therefore, an optimal balance must be struck between efficacy and safety, with careful consideration given to patient characteristics, the type of heterotopic condition, and the potential for adverse events.

Recent developments in the field of molecular radiotherapy and targeted therapy offer exciting opportunities to enhance the efficacy of radiotherapy while reducing its side effects. For instance, the combination of radiotherapy with agents that specifically target the BMP or TGF-β signaling pathways could further inhibit the cellular processes underlying HO [11]. Furthermore, the integration of radio-genomics, which uses genetic profiling to predict patient responses to radiation, may allow for more personalized approaches to treatment [2,3].

Overall, heterotopic pathophysiology represents a complex and multifaceted group of conditions that challenge current medical and therapeutic paradigms. As research into the molecular mechanisms of heterotopy advances, novel and more effective treatments, including radiotherapy, will continue to evolve. Personalized therapeutic approaches, including the combination of radiotherapy with molecularly targeted therapies, hold great promise for improving outcomes in patients suffering from these disorders. Even though RT is a cost-effective treatment, determining radiation dose and fractionation is still an issue, as studies have presented inconclusive data [20,21,22].

This study investigated the impact of heterotopic density variation in radiation dose distribution, calculated using homogeneity and inhomogeneity corrections, on dosimetric and radiobiological parameters in the prophylactic treatment of HO of the hip using external beam radiotherapy. A comparative analysis has been conducted to quantify variations in dose–volume parameters and equivalent biological dose metrics between nominal (homogeneous) plans and accurate inhomogeneity-corrected treatment plans so that future clinical trials can effectively determine the optimum dose for treatment of HO.

## 2. Materials and Methods

### 2.1. Study Design

We reviewed our patient database to identify patients who had received prophylactic RT for the prevention of HO in our institution and then only selected patients whose treatment plans were generated on Eclipse (Eclipse version 16.1, Varian, Palo Alto, CA, USA), a treatment planning system (TPS). Patients whose treatment plans were implemented using the Pinnacle treatment planning system were excluded from the study as the Pinnacle TPS has been decommissioned and removed; hence, these treatment plans were no longer available for review and recalculation. In this retrospective study, a total of 21 patients were included following institutional review board (IRB) clearance. An individual review of each patient’s electronic medical record, regarding both general and radiation therapy (RT), was conducted to collect general and RT data, time intervals between key events, and the timings and doses of RT. The treatment plans of all these patients were originally generated on Eclipse version 16.1.2 (Eclipse, Varian, Palo Alto, CA, USA) without IC before treatment delivery. To investigate the accuracy of the delivered dose, these plans were subsequently recalculated applying the same parameters as the IC-off treatment plans using the analytical anisotropic algorithm (AAA), version 16.1.2, with inhomogeneity correction on. To study the influence of heterotopic density variation, a comparative analysis between the IC-off and -on dose calculations was performed. For this reason, the treatment plans of all patients were recalculated using preset monitor units, employing IC to account for differential radiation absorption and scattering within the treated region, which is known to impact dosimetry significantly [23]. Dose–volume histogram (DVH) data were obtained in Excel format for both the homogeneous and heterogeneous dose distributions to calculate the dosimetric and radiobiological parameters.

### 2.2. Patient Simulation and Radiotherapy Planning

Every patient who was referred underwent computed tomography (CT) simulation in a standardized supine position with appropriate immobilization. Scans, with 3 mm slice thickness, were acquired from the mid-thigh to L3/L4 level for each patient. The clinical target volume (CTV) encompassed the periarticular soft tissues of the hip at risk of ectopic bone formation, based on anatomical guidelines and previous clinical experience. A single fraction was prescribed with a dose of 7 Gy without IC, consistent with established prophylactic regimens. Radiotherapy plans were developed employing a 3D-CRT technique. Patients’ plans were generated using a homogeneous density distribution, i.e., IC off during dose distribution computation for treatment. Antero-posterior beam arrangements were used to achieve the desired distribution profiles, ensuring adequate coverage of the desired volume while minimizing exposure to adjacent organs at risk (OARs).

### 2.3. Dosimetric Evaluation

DVH metrices were extracted for each plan, with IC off and on to assess the quality of the plans. The following dosimetric indices were recorded: maximum dose covering ≤2% of the volume (D_2%_), maximum dose covering ≤50% of the volume (D_50%_), maximum dose covering ≤90% of the volume (D_90%_), maximum dose covering ≤95% of the volume (D_95%_), and maximum dose covering ≤98% of the volume (D_98%_).

To evaluate the quality of the treatment plan, it is essential to precisely calculate the treatment plan indices, such as the dose homogeneity index (HI) and dose gradient index (GI), as defined in ICRU 83 and 91 reports [24,25]. The HI estimates the hot spots within the target and around the target volume [24], whereas the GI estimates the steepness of the dose fall-off within and around the treatment volume [25]. They can be expressed as shown in Equations (1) and (2), where V_50%_ and V_100%_ represent the volumes covered by 50% and 100% dose, respectively.

(1) $$HI=\left(\frac{D_{2\%}-D_{98\%}}{D_{50\%}}\right)$$

(2) $$GI=\frac{V_{50\%}}{V_{100\%}}\;$$

### 2.4. Radiobiological Evaluation

Radiobiological parameters were calculated using equivalent uniform dose (EUD) models incorporating α/β ratios of 3 Gy and 10 Gy, representing late- and early-responding tissues, respectively. Calculations were performed using generalized EUD (gEUD) and linear–quadratic (LQ) model-derived EUD (EUD_LQ) formulations. Additionally, the equivalent dose in 2 Gy fractions (EQD2) was calculated for each α/β scenario under both modeling assumptions. The EQD2 metrics provided a standardized measure of biological effect, enabling intergroup comparisons irrespective of dose fractionation. The *EUD* is defined as the absorbed dose that, if homogeneously delivered to a tumor, produces the same number of tumor cell kills, i.e., produces the same survival fraction of clonogens, as the actual non-homogeneous absorbed dose distribution, as described in other studies [26,27,28]. Clonogen survival is a measure of stochastic magnitude governed by Poisson statistics, and the *EUD* is obtained as an expected value. The phenomenological concept of gEUD was introduced by Niemierko in 1999 [29] to provide a simple formula applicable to both tumors and normal tissue. Its basis is the power law behavior of tissue response to dose, and it has one parameter which is fitted depending on the tissue and the irradiation characteristics, as shown in Equation (3):

(3) gEUD = (∑iviDia)1a

where *v_i_* is the partial volume with absorbed dose *D_i_*, and *a* is a specific parameter that depends on tissue type, treatment modality, dose rate, fraction size, etc. [29]. The gEUD model is based on the Lyman–NTCP concept [30], where EUD is defined as in Equation (4):

(4) $$\mathrm{EUD}\;=\;{\left(\sum_{i}v_{i}D_{i}^{\frac{1}{n}}\right)}^{n}$$

which is similar to the formula in Equation (3). The parameter n = 1/a can be interpreted as converting a non-uniformly distributed dose into an effective dose (EUD) for the entire volume of interest. In Equation (3), when a = 1, the gEUD is the arithmetic mean dose.

The EUD, based on the LQ model [31], is given by

(5) $$\mathrm{EUD}=\frac{-n\alpha }{2\beta }+\frac{n}{2\beta }{\left\{\alpha^{2}-\frac{4\beta }{n}\times ln\left[\sum_{i}^{N}v_{i}\cdot e^{\left(-\alpha \cdot D_{i}-\frac{\beta \cdot D_{i}^{2}}{n}\right)}\right]\right\}}^{1/2}$$

where n is the number of fractions, and *v_i_* is the partial volume with absorbed dose *D_i_*. Hereinafter, this is referred to as LQ_EUD.

Radiobiological assessments included calculating the gEUD for the parameter a = 1 and the EUD_LQ when α/β = 3 Gy and α/β = 10 Gy. Additional radiobiological indices included EQD2, which was also computed for α/β ratios of 3 Gy and 10 Gy, reflecting late and early tissue responses, respectively.

### 2.5. Monte Carlo Data Simulations

Data generation using Monte Carlo (MC) simulations is well known for its superior accuracy in complex situations. The MC simulation produces a probabilistic exploration of possible outcomes. It provides initial conditions that are then perturbed by random variations based on defined probability distributions, leading to a range of potential results. In MC simulations, data are generated by repeatedly running simulations with randomly generated input values, typically based on “baseline data”, allowing for the exploration of a range of possible outcomes and probabilities. 

In this study, we used mean and standard deviation values for different dosimetric and radiobiological parameters to generate simulation data using the MS Excel MC simulation add-in, because in MC simulations, mean and standard deviation are used to define the probability distribution of the simulated data. The mean value represents the center of the distribution, while the standard deviation measures the spread or variability around that center. These values are used to generate random numbers that follow a specific probability distribution, allowing simulations to model uncertain events or phenomena. 

A total of 10,000 random sampling simulation data points were generated to evaluate the dosimetric and radiobiological outcomes of hip irradiation for HO prophylaxis. This large-scale dataset was produced using stochastic sampling techniques that enabled us to model the variability and uncertainties associated with treatment planning parameters, anatomical differences, and dose distributions. The simulated data provided a solid foundation for comprehensive statistical assessment of dose–volume indices and radiobiological metrics under different planning scenarios, with a reliable basis for comparing homogeneous and heterogeneous irradiation strategies. Monte Carlo simulations revealed greater consistency and tighter distributions in homogeneous plans across all parameters, while plans with IC exhibited increased variability and a non-negligible risk of underdosing.

### 2.6. Data Analysis and Statistics

Dosimetric and radiobiological data were reported as mean ± standard deviation (SD) with corresponding 95% confidence intervals (CIs) of the mean values. Since this study had a small sample size, before conducting the comparative statistical analysis, the Shapiro–Wilk test was used, as it can assess whether a dataset follows a normal distribution, which is essential for determining which statistical tests are appropriate to apply. It is particularly effective for small sample sizes. The Wilcoxon signed-rank test, a non-parametric alternative to the paired t-test, was used to compare mean values between the IC-off and -on plans, with a significance level set at *p* < 0.05. The methodology enabled a rigorous comparative assessment of dose uniformity and biological effectiveness in HO prophylaxis, laying the groundwork for optimizing radiotherapy protocols in orthopedic applications.

## 3. Results

### 3.1. Test for Data Distribution Normality and Non-Parametric Paired t-Test

The statistical evaluation presented in the attached Table 1 and Table 2 highlights the impact of inhomogeneity correction on both dosimetric and radiobiological indices. The Shapiro–Wilk test revealed that dosimetric indices, such as D_50%_, D_90%_, D_95%_ (normal distribution), and D_98%_ (deviate from normal distribution), maintained their distribution behavior, while D_2%_, HI, and GI exhibited improved normality following inhomogeneity correction, shifting from non-normal to normal distributions. Conversely, radiobiological indices, which were initially normally distributed, became non-normal post-correction, likely due to the complex, non-linear transformations involved in their computation.

Wilcoxon signed-rank test results further demonstrated statistically significant differences between plans with and without inhomogeneity correction. Except for D_2%_, all dosimetric indices showed significant differences (*p* < 0.05), with large effect sizes indicating strong clinical relevance. Radiobiological indices exhibited consistent and highly significant differences across all metrics, reinforcing the profound influence of inhomogeneity correction on biologically modeled dose outcomes.

Overall, the analysis reveals the necessity of applying inhomogeneity correction during treatment planning, as it substantially affects both physical and biological dose metrics. The results also emphasize the importance of appropriate statistical methods—non-parametric tests in particular—due to deviations from normality, especially in transformed radiobiological data. These findings advocate for rigorous validation of dose evaluation methodologies to ensure accurate, individualized patient care in radiotherapy.

### 3.2. Dosimetric Outcomes

A comparative evaluation of dose distribution indices between homogeneous and inhomogeneity-corrected plans revealed statistically significant differences across all measured parameters (Table 3). As expected, IC-off (homogeneous dose distribution) plans demonstrated superior conformity and uniformity due to their inability to consider actual density variation in the dose distribution computation. Dosimetric data reveal that both techniques achieve similar high-dose regions, with D_2%_ values recorded as 7.30 ± 0.22 Gy (95% CI: 7.20–7.39 Gy) for homogeneous and 7.31 ± 0.16 Gy (95% CI: 7.24–7.38 Gy) for IC plans, showing no statistically significant difference (*p* = 0.6265). However, substantial differences were observed in the median and low-dose regions. D_50%_ decreased from 7.08 ± 0.10 Gy (95% CI: 7.04–7.12 Gy) to 6.87 ± 0.19 Gy (95% CI: 6.79–6.95 Gy) (*p* = 0.0002), D_90%_ from 6.80 ± 14 Gy (95% CI: 6.74–6.86 Gy) to 6.38 ± 0.32 Gy (95% CI: 6.24–6.52 Gy) (*p* = 0.0004), D_95%_ from 6.72 ± 0.18 Gy (95%CI: 6.64–6.79 Gy) to 6.20 ± 0.38 Gy (95%CI: 6.04–6.36 Gy) (*p* = 0.0005), and D_98%_ from 6.60 ± 0.22 Gy (95% CI: 6.51–6.69 Gy) to 6.07 ± 0.39 Gy (95% CI: 5.90–6.24 Gy) (*p* = 0.0011) when transitioning from homogeneous to inhomogeneity-corrected dose distributions.

In terms of dose uniformity, the homogeneous plan demonstrated an overestimation of the dose homogeneity index with an HI of 0.10 ± 0.05 (95%CI: 0.08–0.12) compared to 0.18 ± 0.05 (95%CI: 0.16–0.20) for the IC plan (*p* = 0.0025). Conversely, the GI was more favorable in the IC plan, reflecting a steeper dose fall-off that may enhance sparing of adjacent normal tissues. Specifically, the GI increases from 1.58 ± 0.60 in the homogeneous plan to 2.84 ± 1.32 in the IC plan (*p* = 0.0002).

### 3.3. Radiobiological Metrics

Radiobiological indices (Table 4) further demonstrate overestimation of dose conformity for homogeneous plans compared to IC-on plans, due to a lack of consideration for variable density across the HO region. The gEUD was higher in the homogeneous plan and reduced by 0.22 Gy from 7.02 Gy (7.02 ± 0.09 Gy) to 6.80 Gy (6.80 ± 0.21 Gy) for IC-on plans (*p* = 0.0002). Similar trends were observed with linear–quadratic (LQ) derived EUD values across α/β ratios of 3 Gy and 10 Gy, with all differences reaching statistical significance. For α/β = 3 Gy, EUD LQ was 7.01 Gy (7.01 ± 0.09 Gy) for homogeneous plans and reduced by 0.22 Gy, reached to 6.79 Gy (6.79 ± 0.21 Gy), for IC-on plans (*p* = 0.0002). For α/β = 10 Gy, it reduced by 0.25 Gy from 7.00 Gy (7.00 ± 0.09 Gy) for homogeneous plans to 6.75 Gy (6.75 ± 0.23 Gy) for inhomogeneity-corrected plans (*p* = 0.0002).

Correspondingly, EQD2 values derived from both gEUD and EUD LQ values were higher for homogeneous plans. For instance, EQD2_(α/β=3Gy)_gEUD_ was 14.07 Gy (14.07 ± 0.31 Gy) for homogeneous plans and decreased to 13.35 Gy (13.35 ± 0.70 Gy) for IC-on plans (*p* = 0.0002), with a reduction of 0.72 Gy. EQD2_(α/β=10Gy)_gEUD_ was 9.96 Gy (9.96 ± 0.18 Gy) for homogeneous plans versus 9.53 Gy (9.53 ± 0.41 Gy) for IC-on plans (*p* = 0.0002), reducing by 0.43 Gy. Similarly, EQD2_(α/β=3Gy)_LQ_ reduced by 0.75 Gy from 14.05 Gy (14.05 ± 0.31 Gy) for homogeneous plans to 13.30 Gy (13.30 ± 0.71 Gy) for the IC-on plans (*p* = 0.0002), and EQD2_(α/β=10Gy)_LQ_ reduced from 9.92 Gy (9.92 ± 0.19 Gy) for homogeneous plans to 9.44 Gy (9.44 ± 0.45 Gy) for IC-on plans (*p* = 0.0002), a reduction of 0.48 Gy. These findings show that homogeneous dose distributions overestimate dosimetric consistency and translate into significantly superior biological effectiveness. Also, homogeneous plans deliver sub-optimal doses in terms of potential biological effectiveness, which is realistic as the dose is accurately computed with IC.

### 3.4. Monte Carlo Simulation Data

To evaluate the variability and reliability of dose delivery for HO prophylaxis through hip irradiation, Monte Carlo simulations were conducted, generating 10,000 data points for both homogeneous and inhomogeneity-corrected dose distribution plans. The simulated outcomes were analyzed across four categories of metrics: dosimetric indices, homogeneity and gradient indices, radiobiological parameters, and EQD2. The figures illustrate these results with frequency distributions representing the robustness and spread of each index under both treatment planning conditions.

**Figure 1:**
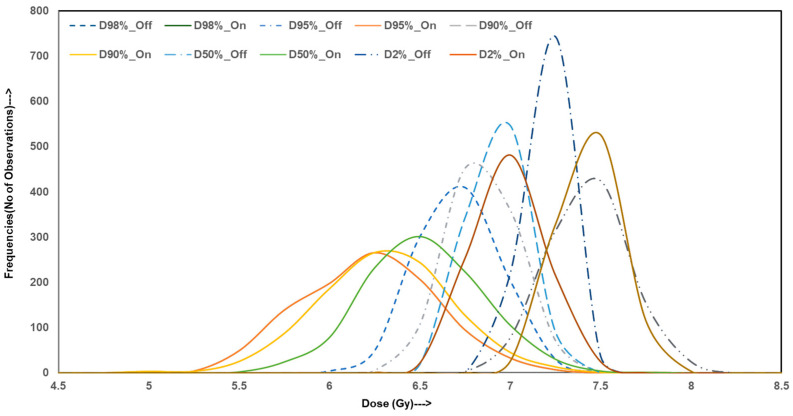
Dosimetric (dose–volume) index curves of Monte Carlo simulation data for IC-off (dotted lines) and IC-on (solid lines) treatment plans for HO hip joint irradiation.

Figure 1 presents the distribution profiles for simulated dose–volume indices including D_2%_, D_50%_, D_90%_, D_95%_, and D_98%_. The distributions for homogeneous plans are tightly clustered around their mean values with minimal spread, indicating high consistency across simulations. In contrast, IC-on distributions display wider variance and greater dispersion from the mean, especially in D_90%_ and D_95%_. These parameters are critical in ensuring adequate target volume coverage. The observed spread in heterogeneous simulations suggests an increased risk of underdosing portions of the target, thereby reducing the reliability of the plan by consistently achieving therapeutic dose levels, which is not the case.

**Figure 2:**
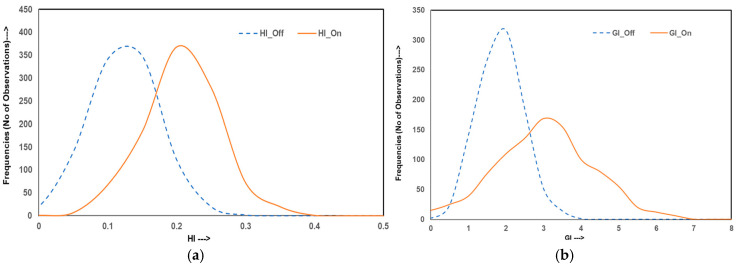
Curves presenting the relationship between Monte Carlo simulation data generated using baseline data for (**a**) HI and (**b**) for GI in IC-off (dotted lines) and IC-on (solid lines) plans for HO hip joint irradiation.

Figure 2a,b illustrate the frequency distribution of the HI and GI, respectively. It is seen that the HI values for homogeneous plans closely cluster around 0.10 with a narrow distribution, falsely reaffirming a high degree of dose uniformity within the target. On the other hand, the IC-on plans exhibit broader HI distributions, centered at higher values (~0.18), highlighting greater internal dose variability. A similar trend is seen in GI distributions that show a narrow spread around 1.58 for IC-off plans, whereas IC-on plans consistently produce a broader GI distribution, clustered above the values of ~2.84, indicative of broad dose fall-offs at the target periphery. While such gradients are disadvantageous for sparing surrounding organs at risk, they are achieved at the expense of uniform dose distribution within the target.

**Figure 3:**
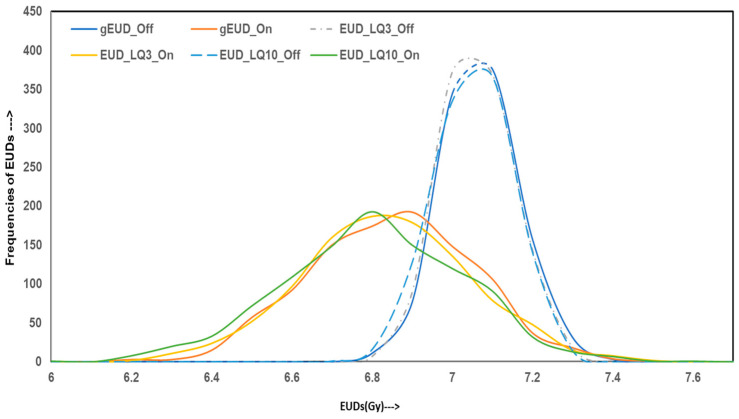
Monte Carlo simulation data of EUDs for IC-off (dotted lines) and IC-on (solid lines) treatment plans for HO hip joint irradiation.

Figure 3 displays the frequency distribution of simulated radiobiological indices: gEUD, LQ-model-based EUD for α/β = 3 Gy and 10 Gy, and their associated EQD2 values. For IC-off plans, gEUD and EUD-LQ distributions are tightly centered around 7 Gy, consistent with the baseline values from the original dosimetric tables. IC-on plans exhibit broader distributions, with a considerable number of cases falling below the 7 Gy threshold. These results suggest that, despite planning conformity, homogeneous distributions deliver suboptimal biological effectiveness in a subset of patients.

**Figure 4:**
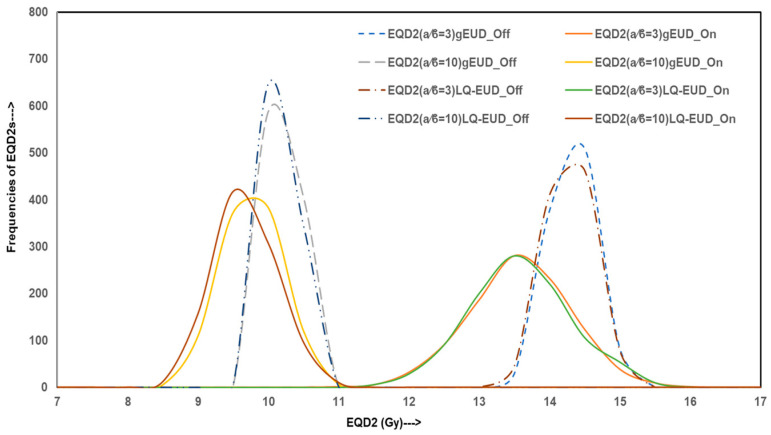
Curves presenting EQD2 relationship for Monte Carlo simulation data for IC-off (dotted lines) and IC-on (solid lines) treatment plans for HO hip joint irradiation.

Figure 4 shows EQD2 values derived from gEUD and LQ-EUD models for both α/β = 3 Gy and 10 Gy. The distributions for IC-off plans cluster strongly around EQD2 values of 14.0 Gy (α/β = 3 Gy) and 9.9 Gy (α/β = 10 Gy), indicating overestimated biologically effective dose delivery, while recomputed IC-on plans demonstrate broader EQD2 distributions with lower mean values (~13.3 Gy and ~9.5 Gy, respectively), confirming reduced radiobiological efficacy in a considerable fraction of simulations. The proportion of simulations falling below the clinically relevant EQD2 thresholds suggests that IC-off plans overestimate dose distribution and may not reliably meet prophylactic dose goals.

These graphical insights emphasize the robustness of dose distributions in HO hip irradiation where Monte Carlo simulation confirms that homogeneous plans overestimate dosimetric uniformity and biological dose reliability due to homogeneous tissue density consideration across the HO region. Hence, the actual dose delivery is far suboptimal. That is why inhomogeneity-corrected plans provide broader dose gradients and reflect real dose distribution across the HO region. The IC-on plans demonstrate inconsistent internal dose distribution and increased likelihood of underdosing, necessitating cautious clinical use of homogeneous plans.

## 4. Discussion

Use of radiation in HO is rare and assessing a large number of patients is often difficult. A multi- institutional approach or clinical trial is needed to gather more patients with clinical follow-up. Such a type of study is warranted and needed. However, this study demonstrates that traditionally practiced homogeneous dose distribution falsely shows adequacy of both dosimetric and radiobiological effectiveness in the prophylactic radiotherapy of HO of the hip. However, this is a poor representation of actual radiation doses since it does not account for density variation in HO region and specifically for high-atomic-number prosthetic devices that are known to affect the dose distribution [23]. The results reveal that IC-off plans optimized for dose uniformity achieve superior outcomes across all critical DVH indices, including D_90%_, D_95%_, and D_98%_. The lower HI in the homogeneous group underscores the enhanced consistency and precision of radiation delivery. These findings align with the previous literature indicating that dose uniformity plays a pivotal role in minimizing treatment failure and reducing the incidence of incomplete prophylaxis; however, it is more critical that dose is computed accurately. In IC-off plans, the dose distribution may be uniform but it is unrealistic and should not be considered.

Many studies have demonstrated the difficulties associated with treatment planning and delivering an accurate dose to patients with hip prostheses. A recent study by Parenica et al. [32] compared the effect of hip prostheses on dose distributions calculated using collapsed cone convolution superposition and Monte Carlo simulations with and without density correction for the implant and surrounding tissues in prostate patients with hip prostheses and concluded that, for the patients with hip prostheses, correct density information for implants and surrounding tissues should be used to optimize the plan and ensure optimal accuracy, i.e., inhomogeneity correction should be used to account for density variation.

The EUD and EQD2 values observed in the homogeneous plans further demonstrate the overestimated clinical superiority of this approach. Notably, on the other hand, IC-on plans yielded lower EUD and EQD2 values than IC-off plans. Given that EUD encapsulates both the magnitude and spatial distribution of the dose, it suggests they have a less potent therapeutic effect, particularly when aiming to suppress the mesenchymal progenitor cell activity that drives ectopic bone formation.

The application of Monte Carlo simulation in hip irradiation for heterotopic ossification (HO) prophylaxis offers a powerful approach to quantify the variability and reliability of treatment planning strategies and provides insight into how statistical uncertainties and inherent anatomical or planning variations can influence the robustness of key dosimetric and radiobiological parameters. The simulated data for homogeneous plans demonstrated narrow distributions with minimal skewness in most parameters, particularly in D_50%_, D_95%_, gEUD, and EQD2 values. This suggests a high degree of reproducibility and reliability in achieving consistent dose coverage of the target region. In contrast, heterogeneous plans displayed broader distributions with increased variance, particularly in D_90%_, D_95%_, and EUD values. These findings align with the baseline data trends, where homogeneous plans exhibited superior homogeneity indices (HIs) and higher generalized equivalent uniform doses (gEUDs).

The broader variability observed in the Monte Carlo data for IC-on plans raises realistic concerns regarding underdosing of sub-volumes within the clinical target. This becomes especially critical in the context of HO prevention, where prophylactic efficacy is highly dose-dependent. Studies such as that of Seegenschmiedt et al. [33] have emphasized that prophylactic irradiation with doses of at least 7 Gy significantly reduces the risk of HO formation following hip arthroplasty. However, none of the HO studies have accounted for inhomogeneity correction. This raises the fundamental question of the actual dose required for the prevention of HO. Our Monte Carlo simulation data reveal that, in heterogeneous plans, a considerable proportion of the distribution tail falls below this threshold, especially in terms of D_95%_ and gEUD values. This variability could compromise the prophylactic success rate, particularly in high-risk patients undergoing hip arthroplasty.

Moreover, the EQD2 values derived from the simulated gEUD data were consistently lower in IC-on plans reinforcing the concern about radiobiological effectiveness. For example, while homogeneous plans maintained mean EQD2 (α/β = 3 Gy) values close to 14 Gy across simulations, recalculated IC-on plans frequently exhibited simulated EQD2 values falling below 13 Gy. The potential clinical implications of these discrepancies are supported by earlier findings from Hu et al. [34], who noted that even modest reductions in biologically effective dose (BED) may influence HO recurrence rates post-operatively.

When benchmarked against simulation-based treatment studies by Park and Kung [35] and Chetty et al. [36], our findings emphasize the importance of robust but accurate dose modeling in anatomically complex regions such as the pelvis. These authors reported that AAAs can underestimate the impact of dose inhomogeneity, particularly when relying on analytical approximations. Monte Carlo-based simulation provides a more accurate reflection of dose computation, especially in the context of soft tissue–bone interfaces where scatter and absorption differ significantly and create significant dose perturbation [23].

Interestingly, the GI consistently favored the IC-on plans in both baseline and simulated datasets. This supports the notion that IC-on plans may offer better sparing of adjacent normal tissues, such as the bladder and rectum. This observation aligns with findings by Pederson et al. [37], who advocated for sharper dose gradients in pelvic radiotherapy to reduce late toxicity. However, this benefit must be balanced against the increased uncertainty and lower dose conformity to the target volume, particularly in post-operative settings where precise prophylaxis is critical.

The simulation also highlights the need for individualized treatment planning. The increased variance in the inhomogeneity-corrected plans suggests that patient-specific anatomical variability could substantially influence treatment outcomes. Incorporating adaptive planning strategies or robust optimization techniques may help mitigate these uncertainties [38]. Monte Carlo simulations underscore the clinical stability and radiobiological robustness of homogeneous dose distribution for HO hip irradiation. Although IC-on planning offers theoretical advantages in tissue sparing, the simulations reveal a greater susceptibility to target underdosing. These findings, supported by both baseline and simulated data, indicate that accurate dosimetry with IC should be undertaken. In the context of accurate dose distribution and lack of actual dose information, IC doses should be provided for the prevention of HO.

## 5. Conclusions

Traditional dose distribution without inhomogeneity correction is generally accepted, where a nominal dose of 7 Gy in a single fraction is used for HO. The actual dose depends on the patient’s geometry and the prosthetic device. However, with advanced radiation dose calculation algorithms, IC-on dose computation for HO dosimetry reveals a non-uniform dose distribution, and its impact in radiobiological modeling is presented. Even though the uncorrected doses seem uniform, they are unrealistic and should be discarded. Additional data are needed for dose and fractionation with respect to the actual dose delivered to the sites affected by prosthetic devices.

## Figures and Tables

**Table 1:** Shapiro–Wilk test results to validate normality of dosimetric indices of IC-off and IC-on plans.

Radiobiological Indices	Inhomogeneity-Correction-Off Plans						Inhomogeneity-Correction-On Plans					
	W (Test)	Skew	ES	*p*-Value	H0	Norm	W (Test)	Skew	ES	*p*-Value	H0	Norm
						Y/N						Y/N
gEUD	0.940	−0.449	0.181	0.222	NR	Y	0.887	0.502	0.239	0.020	R	N
EUD_(α/β=3)_LQ_	0.938	−0.574	0.171	0.199	NR	Y	0.883	0.495	0.241	0.017	R	N
EUD_(α/β=10)_LQ_	0.921	−0.922	0.200	0.091	NR	Y	0.882	0.463	0.216	0.016	R	N
EQD2_(α/β=3)_gEUD_	0.942	−0.415	0.179	0.236	NR	Y	0.885	0.521	0.241	0.018	R	N
EQD2_(α/β=10)_gEUD_	0.941	−0.425	0.179	0.232	NR	Y	0.886	0.515	0.240	0.019	R	N
EQD2_(α/β=3)_LQ_	0.940	−0.539	0.169	0.215	NR	Y	0.881	0.515	0.243	0.015	R	N
EQD2_(α/β=10)_LQ_	0.923	−0.893	0.198	0.100	NR	Y	0.880	0.478	0.218	0.015	R	N

Note: W (Test) is the statistic reference value from the Shapiro–Wilk test table for a sample size of 21 and α = 0.05 and is equal to 0.908. Abbreviations: W (Test) = Test statistic value; Skew = Skewness; ES = Effect Size; *p*-value = *p*-value for data normality; H0 = H0 hypothesis; Norm = data normality; R = Rejected; NR = Not Rejected; Y = Yes; N = No.

**Table 2:** Shapiro–Wilk test results to validate normality of radiobiological indices of IC-off and IC-on plans.

Dosimetric Indices	Inhomogeneity-Correction-Off Plans						Inhomogeneity-Correction-On Plans					
	W (Test)	Skew	ES	*p*-Value	H0	Norm	W (Test)	Skew	ES	*p*-Value	H0	Norm
						Y/N						Y/N
D_2%_	0.859	1.115	0.214	0.006	R	N	0.918	1.032	0.172	0.078	NR	Y
D_50%_	0.932	−0.497	0.120	0.154	NR	Y	0.950	0.054	0.181	0.333	NR	Y
D_90%_	0.944	−0.505	0.141	0.257	NR	Y	0.945	0.043	0.150	0.276	NR	Y
D_95%_	0.936	−0.742	0.180	0.182	NR	Y	0.930	0.191	0.141	0.139	NR	Y
D_98%_	0.907	−1.274	0.160	0.048	R	N	0.885	0.520	0.226	0.018	R	N
HI	0.881	1.008	0.156	0.015	R	N	0.911	−0.570	0.146	0.056	NR	Y
GI	0.618	2.919	0.304	3.10 × 10^−6^	R	N	0.918	0.572	0.204	0.079	NR	Y

Note: W (Test) is the statistic reference value from the Shapiro–Wilk test table for a sample size of 21 and α = 0.05, and is equal to 0.908. Abbreviations: W (Test) = Test statistic value; Skew = skewness; ES = effect size; *p*-value = *p*-value for data normality; H0 = H0 hypothesis; Norm = data normality; R = rejected; NR = not rejected; Y = yes; N = no.

**Table 3:** Dosimetric, plan uniformity, and dose gradient parameters for IC-off and IC-on treatment plans for HO hip joint irradiation.

Dosimetric Indices	Inhomogeneity-Correction-Off Plans		Inhomogeneity-Correction-On Plans		*p*-Value
	Mean ± SD (Gy)	95%CI	Mean ± SD (Gy)	95%CI	
D_2%_	7.30 ± 0.22	7.20–7.39	7.31 ± 0.16	7.24–7.38	0.6265
D_50%_	7.08 ± 0.10	7.04–7.12	6.87 ± 0.19	6.79–6.95	0.0002
D_90%_	6.80 ± 0.14	6.74–6.86	6.38 ± 0.32	6.24–6.52	0.0004
D_95%_	6.72 ± 0.18	6.64–6.79	6.20 ± 0.38	6.04–6.36	0.0005
D_98%_	6.60 ± 0.22	6.51–6.69	6.07 ± 0.39	5.90–6.24	0.0011
HI	0.10 ± 0.05	0.08–0.12	0.18 ± 0.05	0.16–0.20	0.0025
GI	1.58 ± 0.60	1.32–1.84	2.84 ± 1.32	2.27–3.40	0.0002

**Table 4:** Radiobiological parameters for inhomogeneity-uncorrected and inhomogeneity-corrected treatment plans for HO hip joint irradiation.

Radiobiological Indices	Inhomogeneity-Correction-Off Plans		Inhomogeneity-Correction-On Plans		*p*-Value
	Mean ± SD (Gy)	95%CI	Mean ± SD (Gy)	95%CI	
gEUD	7.02 ± 0.09	6.98–7.06	6.80 ± 0.21	6.71–6.89	0.0002
EUD_(α/β=3)_LQ_	7.01 ± 0.09	6.98–7.05	6.79 ± 0.21	6.70–6.88	0.0002
EUD_(α/β=10)_LQ_	7.00 ± 0.09	6.96–7.04	6.75 ± 0.23	6.66–6.85	0.0002
EQD2_(α/β=3)_gEUD_	14.07 ± 0.31	13.93–14.20	13.35 ± 0.70	13.05–13.65	0.0002
EQD2_(α/β=10)_gEUD_	9.96 ± 0.18	9.88–10.04	9.53 ± 0.41	9.35–9.71	0.0002
EQD2_(α/β=3)_LQ_	14.05 ± 0.31	13.92–14.18	13.30 ± 0.71	13.00–13.61	0.0002
EQD2_(α/β=10)_LQ_	9.92 ± 0.19	9.84–10.00	9.44 ± 0.45	9.24–9.63	0.0002

## Data Availability

Data are available upon request.

## Abbreviations

The following abbreviations are used in this manuscript:

HO	Heterotopic ossification
IC	Inhomogeneity correction
BMP	Bone morphogenetic protein
Wnt	Wingless-related integration site
MSC	Mesenchymal stem cell
DVH	Dose–volume histogram
CI	Conformity index
HI	Homogeneity index
GI	Gradient index
LQ	Linear–quadratic model
EUD	Uniform equivalent dose
gEUD	Generalized uniform equivalent dose
EQD2	Equivalent dose in 2 Gy fractions
BED	Biologically effective dose
